# scRNA-seq analysis discovered suppression of immunomodulatory dependent inflammatory response in PMBCs exposed to silver nanoparticles

**DOI:** 10.1186/s12951-024-02364-0

**Published:** 2024-03-17

**Authors:** Haribalan Perumalsamy, Xiao Xiao, Hyun-Yi Kim, Tae-Hyun Yoon

**Affiliations:** 1 Center for Creative Convergence Education, Hanyang University, Seoul, 04763 Republic of Korea; 2 Institute of Next Generation Material Design, Hanyang University, Seoul, 04763 Republic of Korea; 3 Department of Chemistry, College of Natural Sciences, Hanyang University, Seoul, 04763 Republic of Korea; 4 NGeneS Inc, 362, Gwangdeok 1-ro, Sangnok-gu, Ansan-si, Gyeonggi-do 15495 Republic of Korea; 5 Department of Medical and Digital Engineering, Hanyang University, Seoul, 04763 Republic of Korea; 6 Research Institute for Convergence of Basic Science, Hanyang University, Seoul, 04763 Republic of Korea

**Keywords:** AgNPs, scRNA-seq analysis, Toxicity, Cellular-metal ion association, Monocytes

## Abstract

The assessment of AgNPs toxicity in vitro and in vivo models are frequently conflicting and inaccurate. Nevertheless, single cell immunological responses in a heterogenous environment have received little attention. Therefore, in this study, we have performed in-depth analysis which clearly revealed cellular-metal ion association as well as specific immunological response. Our study didn’t show significant population differences in PMBC between control and AgNPs group implying no toxicological response. To confirm it further, deep profiling identified differences in subsets and differentially expressed genes (DEGs) of monocytes, B cells and T cells. Notably, monocyte subsets showed significant upregulation of metallothionein (MT) gene expression such as *MT1G*, *MT1X*, *MT1E*, *MT1A*, and *MT1F.* On the other hand, downregulation of pro-inflammatory genes such as *IL1β* and *CCL3* in both CD16 + and CD16- monocyte subsets were observed. This result indicated that AgNPs association with monocyte subsets de-promoted inflammatory responsive genes suggesting no significant toxicity observed in AgNPs treated group. Other cell types such as B cells and T cells also showed negligible differences in their subsets suggesting no toxicity response. Further, AgNPs treated group showed upregulation of cell proliferation, ribosomal synthesis, downregulation of cytokine release, and T cell differentiation inhibition. Overall, our results conclude that treatment of AgNPs to PMBC cells didn’t display immunological related cytotoxicity response and thus motivate researchers to use them actively for biomedical applications.

**Supplementary Information:**

The online version contains supplementary material available at 10.1186/s12951-024-02364-0.

## Introduction

Silver nanoparticles (AgNPs) are the most commercialized metals and have wide range of biomedical applications, including pharmaceuticals, cosmetics, biosensing, bioimaging, and drug delivery [1–6]. In spite of having many beneficial effects in microbiological, health systems, and consumer spheres, it is not classified in the European Regulation on Chemical Substances (REACH) [7]. The modulation of the immune response by AgNPs is of particular importance since uncontrolled immunological activation or suppression can result in allergy reactions as well as malfunctioning of the body’s immune response to damage, infection, and cancer [8, 9]. At present, the role of AgNPs in modulating the immune response is extremely novel and therefore, understanding their toxicity related immunological response is crucial for designing highly effective eco-friendly nanomaterials.

Monocytes are immune cells that participate in innate immunity, the first line of immune defense when they confront nanoparticles. Whereas, B cells are activated by ligand interaction or external stimuli, which initiates intracellular signaling and leads to the internalization of antigen for processing and presentation to T cells [10]. Exposure of AgNPs can elevate metallothionein’s expression which plays an important role in regulating reactive oxygen species (ROS) and preventing oxidative stress [11]. Although malfunctioning of immune response has been linked to many human diseases, their action differs depending on the concentration of nanoparticles. For instance, when monocytes recognize higher concentration of nanoparticles results in death process whereas, low concentration of nanoparticles can activate monocyte to induce inflammatory immune response [12]. Similarly, the release of cytokines must be tightly regulated to thwart overstimulation of the immune response. Previous study showed that AgNPs stimulated immune reactions at sub lethal concentrations by increasing interleukin (IL) induction, and ROS [12]. For example, IL-1β is one of the pro-inflammatory cytokine that plays a vital role in both innate and systemic immune response upon inflammasome activation [13]. Therefore, controlled, and rapid initiation and effective resolution of inflammation is vital for normal homeostasis maintenance.

Many approaches on toxicity assessment of AgNPs have been previously studied using in vitro or in vivo analysis. For instance, Vrcek et al. demonstrated the dose-dependent toxicity (0.5 and 50 mg L^− 1^) of AgNPs for 3 h in human hepatoblastoma cells which showed clear toxic effects when cells exposed to higher concentration, and other study established the decrease in cell viability, membrane integrity, and increased ROS generation in human hepatocarcinoma and mononuclear cells upon AgNPs treatment for 24 h indicating that AgNPs are toxic [14]. In case of in vivo analysis, > 12.5, 25 and 50 µg/mL concentrations of AgNPs exhibited pro-inflammatory cytokines response and loss of male and female germ cells in mice group [15]. Additionally, Kobos et al. also showed 2 mg/kg AgNPs influenced pro-inflammatory cytokines as well as histopathological changes in mice. AgNPs are also proven to exhibit defects in spinal cord, heart, and eye [16].

Furthermore, there has been limited study on ex vivo toxicity evaluation, notably on human primary peripheral blood mononuclear cells (hPBMCs), which could be a feasible alternative approach to study leukocytes population [17, 18]. hPBMC belong to heterogeneous cell types which serve as a crucial ex vivo model to stimulate biological response after blood exposure to AgNPs, but such heterogeneity could be masked by bulk assays [19]. However, there were few studies on cellular interactions between AgNPs and hPBMCs, most of them were analyzed using bulk methodological approaches, and due to an unsophisticated assumption that the hPBMCs are homogeneous. For example, the oxidative burst and DNA damage of hPBMCs exposed to AgNPs were analyzed [20] by bulk methodological approaches (H2DCFDA assay, comet assay). Likewise, Pourhoseini et al. measured the average biological uptake of AgNPs in hPBMC using coupled plasma mass spectrometer [21]. Nevertheless, hPBMCs consist of different cell types, each of them could respond with quite different behaviors. Therefore, performing data analysis at the single-cell level to study the potential effects of AgNPs to visualize cellular response in a heterogenous environment is highly desirable and poorly investigated.

The primary research topic here is whether AgNPs produce particular immunomodulatory responses upon contact with various peripheral blood mononuclear cells (hPBMCs) at the single cell level. Our findings will help us better understand how AgNPs stimulate immune responses following exposure and may be employed safely.

## Materials and methods

### Preparation and characterization of AgNPs

The detailed information about sample preparation and characterization was reported previously [22]. Briefly, whole blood was drawn from healthy donors as they can mimic real-life experiments and triplicate measurements were performed with blood obtained from different donors for each replication. AgNPs coated with branched-polyethylenimine (bPEI) with a nominal core diameter of 40 nm (denoted as bPEIAg40), purchased from NanoComposix (San Diego, CA, USA). The hPBMCs samples were treated with 2 µg/mL of AgNPs in RPMI complete medium for 3 h at 37°C and 5% CO_2_. The dissolution of bPEIAg40 NPs in deionized (DI) water and RPMI media for 3 and 24 hours was studied using inductively coupled plasma-mass spectrometry (ICP-MS). The physicochemical properties of AgNPs was measured including core size (37 ± 4 nm), hydrodynamic size (176 ± 2 nm), and zeta potential in RPMI media (-11.7 ± 1.8 mV) [22]. Our previous study also reported that AgNPs cellular association greatly differs depending on surface coatings and particle size [23]. Thus, 40 nm was chosen to stimulate signaling responses of immune cells as NPs shown to exhibit size-dependent cytotoxicity. Moreover, it was also reported that AgNPs, particle size of 50 nm or above demonstrated to have least effect on cell viability and signaling response [24, 25].For gel beads-in-emulsion (GEM) creation and barcoding, single cell 3’ v3.1 gel beads were employed, which are comprised of a master mix with cell surface protein labels and partitioning oil added to a chromium chip. Following the manufacturer’s instructions (Next GEM single cell kit V3.1, Pleasanton, CA, USA), the cDNA amplification and post GEM-RT cleanup processes were completed satisfactorily. Paired end readings were used for sequencing on the Illumina HiSeq2500. A depth of between 50,000 and 70,000 reads per cell was achieved by sequencing about 2000 cells per sample.

### Data acquisition, quality control and preprocessing

The high-dimensional, large unbiased scRNA-seq data from two conditions, control and treated, were obtained separately using CellRanger (Cell Ranger, version v4.0.0, 10x Genomics). The resulting count matrices were combined to perform downstream analysis using Seurat v4.3.0 [26]. Quality control measures were applied to the integrated dataset using Seurat’s standard workflow [27]. Cells with low gene detection rates (200 genes) or strong mitochondrial gene expression (> 10% mitochondrial gene content) were eliminated as outliers or low-quality cells. Gene expression values were log-transformed and normalized using the “LogNormalize” method in Seurat, ensuring that the expression distributions were comparable across cells. For integration of datasets, anchors corresponding across datasets were identified and were used to guide dataset integration as described [26].

### Dimensionality reduction, clustering, and cell annotation

Principal component analysis (PCA) was performed on the normalized expression matrix to reduce the dimensionality of the data [28]. The 30 principal components capturing the most significant sources of variation were selected based on their corresponding eigenvalues. The selected principal components were used as an input for clustering analysis using the Seurat FindNeighbors and FindClusters functions, which employ shared nearest neighbor (SNN) modularity optimization [29]. The resolution parameter was set to 0.5 to define the granularity of the clusters. To assign cell type identities to the clusters, singleR package [30] was utilized. We classified each cell from a different sample group in our datasets as attributed to one of the 10 major cell types, followed by one of the 29 cell subtypes. The major cell type assignments could be used for comparison with other transcriptomic studies, while the cell subtype assignments could provide more in-depth information on the cellular responses. After the cells were assigned, the cell population changes between AgNPs treatment and untreated groups were analyzed respectively.

### Uniform manifold approximation and projection (UMAP) visualization

We used uniform manifold approximation and projection visualization (UMAP) projection to visualize the high-dimensional scRNA-seq data to qualitatively observe the transcriptomic changes by AgNPs exposure. Each cell in the UMAP plot was assigned a location on a two-dimensional map. The changes in the location of the dots intuitively demonstrated the impact of AgNPs exposure. The integrated Seurat object was processed using the “RunPCA” (npcs = 30) and “RunUMAP” (dims = 1:20) functions. The “Dimplot” function made it easy to label the UMAP plots with cell types. First, we created an overall UMAP plot of all cells with major cell types labeled. Then, we established a UMAP plot with cell subtype labels for each major cell type.

### Differential expressed genes (DEGs) and gene ontology (GO) enrichment analysis

Differentially expressed genes (DEGs) between the treated and control groups were identified using the DESeq2 v1.36.0 [31]. Genes were considered significant if their adjusted p-values (FDR) were below the threshold of 0.05. To gain functional insights into the identified DEGs, gene ontology (GO) enrichment analysis were performed using the clusterProfiler v4.4.4 [32]. Multiple testing correction (Benjamini-Hochberg procedure) was applied and GO terms with adjusted p-values below 0.05 were considered significantly enriched.

### String network analysis

String network analysis was employed to investigate how the identified DEGs may interact with one another (https://string-db.org/). The string software has a large database that documents gene interactions. Gene fusion, gene co-expression, co-mentioned in a paper abstract, and other interactions are examples of interactions. The string software will assign a score (0–1) to each pair of genes based on the database and certain scoring rules. The score represents the level of confidence in the interactions, with 1 representing the highest level of confidence. Therefore, during string network analysis, we set the threshold to 0.4.

### Statistical analysis

For all DEG analyses, a significant change was defined as an adjusted p-value (Benjamini-Hochberg analysis) less than 0.05 and a fold change over two times. For all GO and GESA analyses, an adjusted p-value (Benjamini-Hochberg analysis) of less than 0.05 was used to identify significant enrichment.

## Results

### Cell annotation and cell types identification

**Fig. 1:**
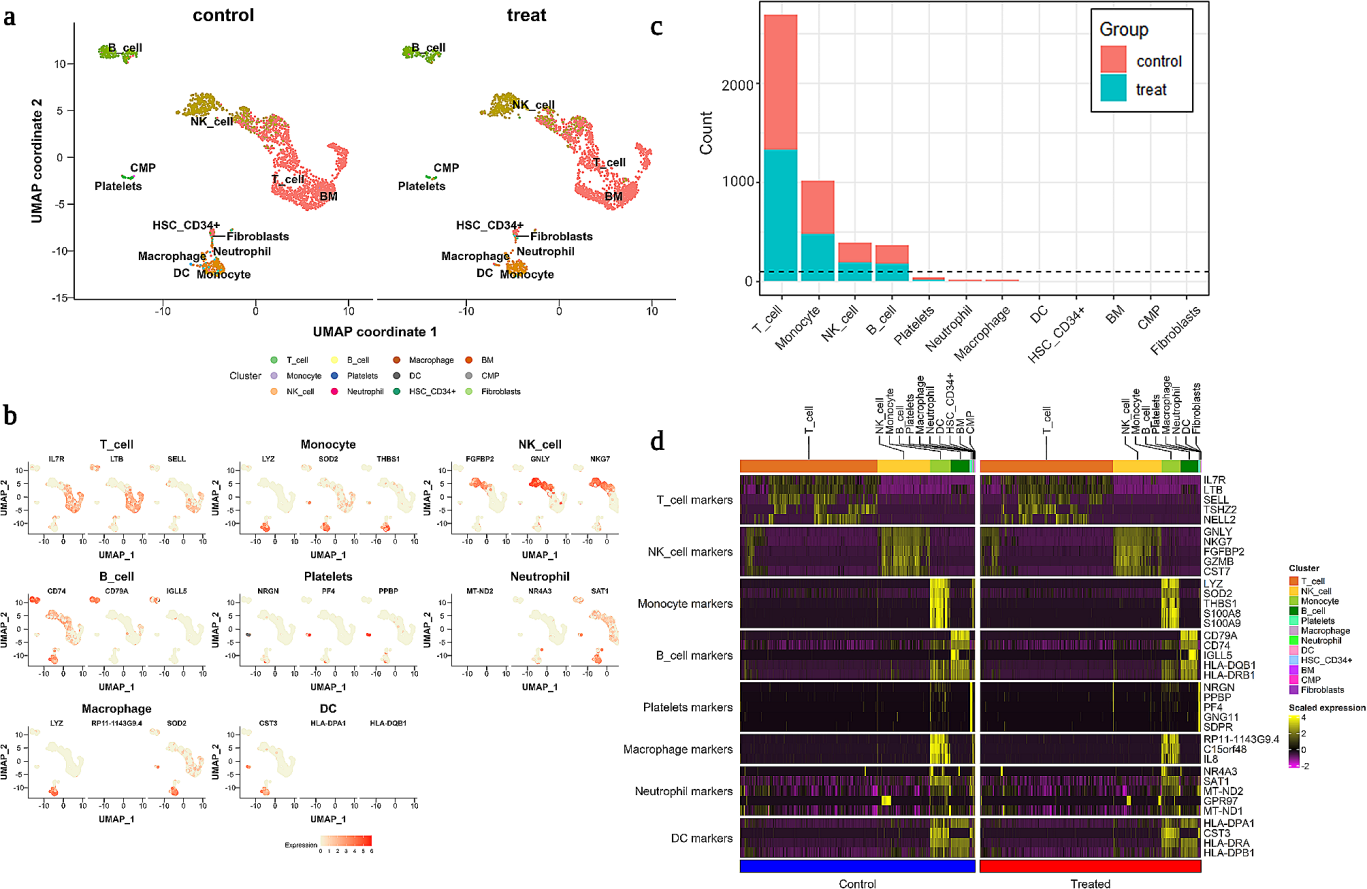
Population and transcriptomic profile analysis of the major cell types. (**a**) Uniform manifold approximation and projection (UMAP) of single-cell transcriptomes of control group and treatment group (AgNP treated). (**b**) Cell population of all cell types, expressed as number of leukocytes. (**c**) Expression of reference cell markers for major cell types. (**d**) Overview of gene expression pattern between control and treatment groups

In this study, initially, we compared the cellular population differences between control and AgNPs treated group (Figure S1a) and we performed SNN clustering and identified more than 11 distinguished heterogeneity clusters based on their marker’s genes expression (Figure S1b). Furthermore, our cell annotation analysis revealed major immune cell types including monocytes, B cells, T cells, NK cells, macrophages, neutrophils, and dendritic cells based on reference marker gene expression, and visualized in UMAP plot (Fig. 1a). The expression of the reference marker genes was listed and visualized using the UMAP plot (Fig. 1b), and we were able to confirm the cells were rationally annotated using these expression plots since certain cell markers only show high expression within the corresponding major cell types. Later, we compared the population differences of each cell type between the groups and visualized in bar plot which was distinguished by unique colors (Fig. 1c). However, AgNPs at a dose of 2 µg/mL didn’t cause obvious changes in the major immune population, indicating that there was no significant toxicity. But we speculated that after 2 µg/mL AgNPs exposure, the cellular expression on gene level might differ with each immune cell type. Therefore, heatmap was performed to identify expression of selected marker genes (listed in Fig. 1d) between treated and untreated groups. As a result, we have discovered changes in the gene expression that needed spotlight. Henceforth, we divided each major cell type into more specific subgroups to perform DEGs and GO analysis for these subgroups.

### Upregulation of metallothionein genes and downregulated inflammatory genes in monocytes upon AgNPs exposure

**Fig. 2:**
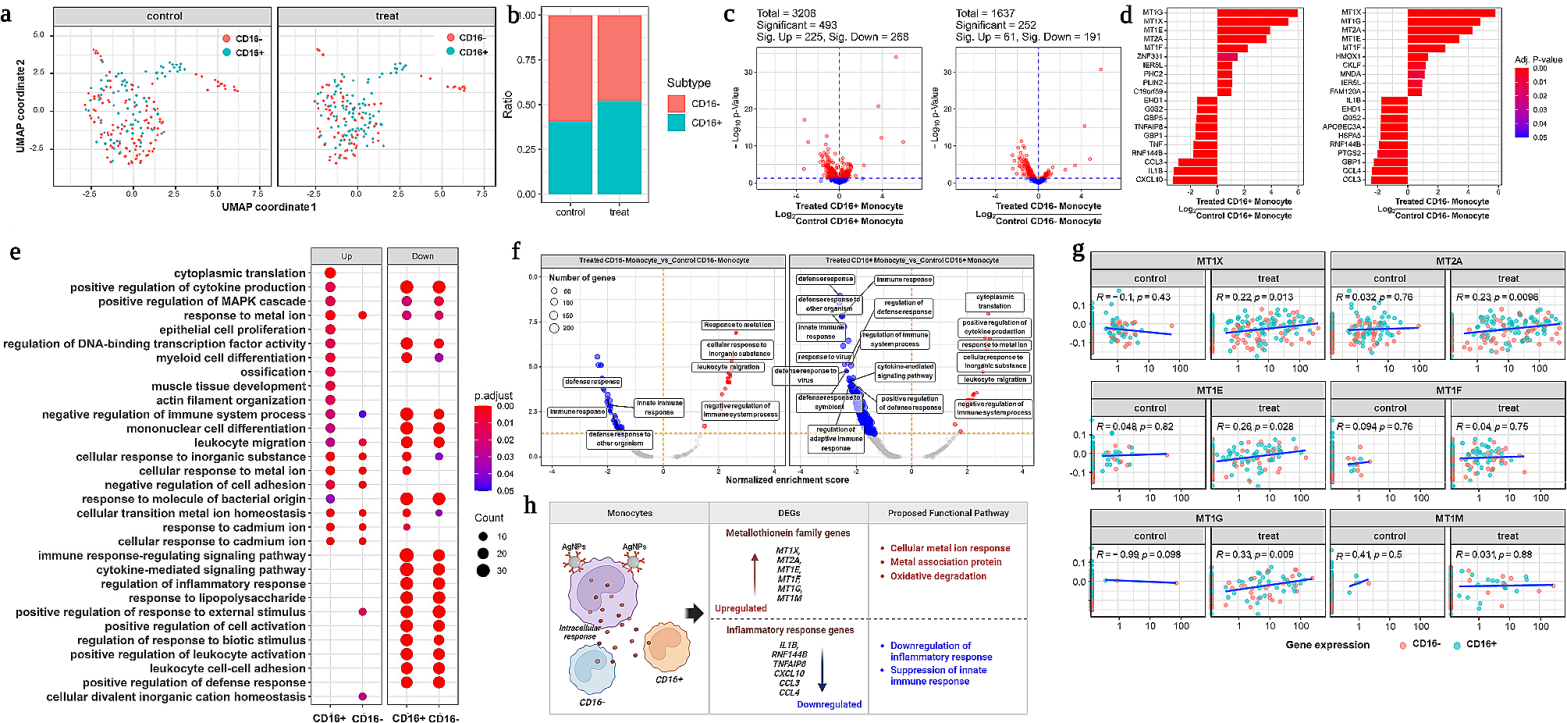
DEGs and Enrichment analysis of Monocytes. (**a**) Monocyte subsets from the control and treated groups are plotted using UMAP. (**b**) Population differences of monocyte subsets from untreated control and treated group. (**c**) Volcano plot showed significant gene expression from both the control and treated groups. (**d**) Bar plot graph showed differentially expressed genes (DEGs) from monocyte subsets. (**e**) Gene ontology enrichment analysis showed up and downregulation genes involved in various functional annotation of pathway list. (**f**) Gene set enrichment analysis (GSEA) of significant genes from monocyte subsets. (**g**) Types of metallothionein genes are mostly involved in metal ion –cellular responses. (**h**) Illustration of monocytes and their subsets with their up and down regulated genes involved in proposed functional pathway. For all DEG analyses, a significant change was defined as an adjusted p-value (Benjamini-Hochberg analysis) less than 0.05 and a fold change over two times. For all GO and GESA analyses, an adjusted p-value (Benjamini-Hochberg analysis) of less than 0.05 was used to identify significant enrichment

Monocytes and their subtypes were identified after AgNPs exposure compared with untreated group. Identified monocyte subsets, CD16 + and CD16- might contribute to heterogeneity in cellular response, as shown in UMAP plot of monocytes (Fig. 2a**)**. The population ratio of the two monocyte subsets were compared and identified that the treated group’s CD16 + population showed non-significant increase compared to the untreated group, while the CD16- population showed no significant decrease population (Fig. 2b). The volcano plots (Fig. 2c) from CD16 + and CD16- monocytes were used to visualize DEGs expression in both the treated and untreated groups, and the red-colored dots denote significant DEGs (adjusted p-value less than 0.05). The CD16 + monocyte subset discovered 3208 DEGs in total, out of which 493 were significantly de-regulated, with 225 being upregulated and 268 being downregulated. In the case of the CD16- monocyte subset, 1637 DEGs were discovered in total, out of which 252 exhibited significant differences, with 61 upregulated and 191 downregulated (Fig. 2c).

The significant DEGs from each monocyte subsets were displayed in a bar plot graph, indicating the up and down regulation of genes from both treated and untreated groups. (Fig. 2d). Interestingly, we identified metallothionein-related genes (MT) from both monocytes, particularly *MT1G, MT1X, MT1E, MT2A, MT1F*, and *MT1M* were significantly upregulated upon treatment (Fig. 2d), indicating that these may contribute to maintain physiological balance and regulate immune homeostasis [33]. In contrast, inflammatory cytokines such as *IL1B*, *RNF144B*, and *TNFAIP8* as well as chemokines including *CXCL10*, *CCL3*, and *CCL4* were substantially downregulated (Fig. 2d). The functional heterogeneity of CD16 + and CD16- monocyte subsets showed DEGs that were visualized in Fig. 2d was similar trend in MT related genes as well as inflammatory responsible genes. However, CD16- monocytes differed slightly from CD16 + monocytes based on adjusted p_value of the MT genes and upregulation of *HMOX1* genes (Fig. 2d) respectively.

Further, we performed GO enrichment analysis to visualize biological, cellular, and molecular function of significant genes from monocytes. As a result, we identified that the upregulated genes from CD16 + monocyte subset mostly involved in cytoplasmic translation, regulation of cytokine production, cellular response to metal ion, and epithelial cell proliferation etc. (Fig. 2e). Particularly, most of the MT genes (*MT1G, MT1X, MT1E, MT2A, MT1F*, and *MT1M*) involved in metal ion-cellular response, transport, homeostasis, and metallopeptidase activity. Compared to the CD16 + monocyte subset, there is only minimal number of genes involved in positive regulation of external stimulus and cation homeostasis was observed in CD16- (Fig. 2e). However, both the monocyte subsets actively participated in downregulation of cellular immunological response followed by cellular association of metal-ion (Figure S2c). Especially, inflammatory cytokine and chemokine-related genes were significantly downregulated due to AgNPs exposure (Figure S2d), indicating downregulation of innate and adaptive immune response, positive regulation of cytokine production, leukocyte migration, cell cycle (Figure S2e) and regulation of inflammatory defense signaling pathways.

Further, gene set enrichment analysis (GSEA) was used to compare statistically significant and concordant differences between the treated and control groups using normalized enrichment score analysis. Figure 2f depicted the number of genes from each monocyte subsets involved in concordant responses of the normalized enrichment score of the AgNPs-treated group. Most of the genes from CD16 + monocyte showed active involvement compared to CD16- by downregulating various biological, cellular, and molecular functional aspects including innate/adaptive defense response, regulation of immunological response, cytokine mediated signaling response, and so on (Fig. 2f). In contrast, the upregulated genes in either monocyte subset may be actively involved in cellular response to metal ions, leukocyte migration, negative regulation of immune system response and cytoplasmic translation respectively (Fig. 2f).

Downregulation of DEGs from both CD16 + and CD16- monocyte subsets significantly reduced immunological responses including defense and other immunological responses. In a rank of an ordered dataset, the defense response showed a negative enrichment score for both CD16+ (-0.47; p-value < 0.001) and CD16- (-0.40; p-value < 0.001). Additionally, similar trends in immunological response were noticed in both CD16+ (-0.457; p-value < 0.001) and CD16- (-3.8; p-value < 0.001) of rank in ordered dataset (Figure S2f). Furthermore, the upregulated genes from both CD16 + and CD16- monocyte subsets showed active involvement with positive enrichment scores in response to metal ion (0.937; p-value < 0.001), cellular transition metal ion homeostasis (0.82; p-value < 0.001), cytoplasmic transition (0.789; p-value < 0.001) and leukocytes migration (0.882; p-value < 0.001) (Figure S2f).

Upon AgNPs treatment, we observed cellular association mediated upregulation of MT indicating the influence of cellular response in monocytes (Fig. 2g). More than six genes, including *MT1X, MT2A, MT1E, MT1F, MT1G*, and *MT1M*, demonstrated significant cellular association and responses in AgNPs-treated groups compared to untreated groups. However, downregulation of pro-inflammatory cytokines and chemokines suppressed further inflammatory responses caused by upregulated MT genes. This indicated that AgNPs may be closely associated with monocytes, helping in the removal of cytotoxic effects as well as early acute inflammatory responses by upregulating MT genes (Fig. 2h).

### B cells-AgNPs initiated cell proliferation and suppresses B lymphocyte proliferation

**Fig. 3:**
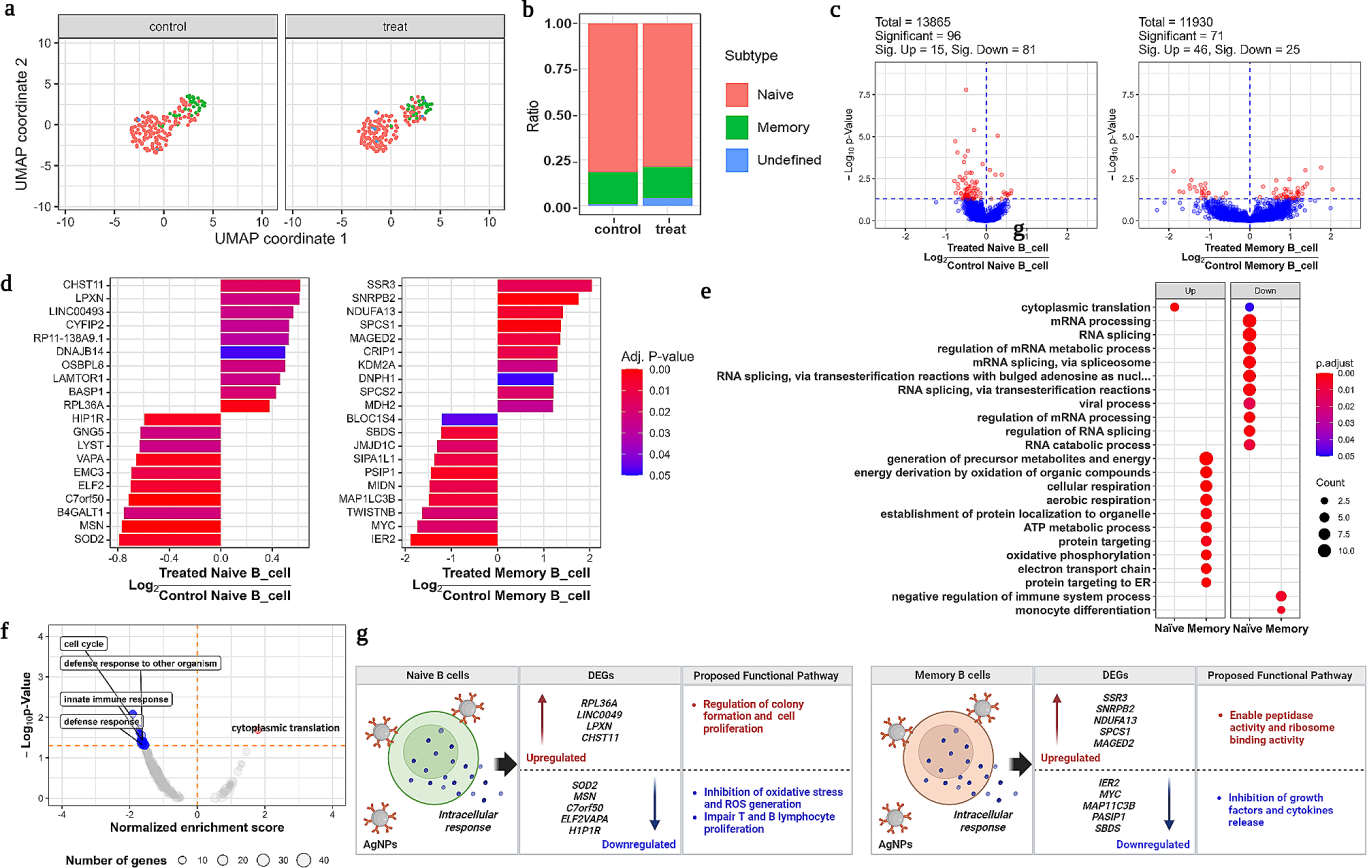
DEGs and Enrichment analysis of B cells. (**a**) UMAP is used to plot B cell subsets from the control and treated groups. (**b**) Population differences of B cells subsets from untreated control and AgNP treated group. (**c**) Significant gene expression from both the control and treated groups was visible on the volcano plot. (**d**) Differentially expressed genes (DEGs) from B cell subsets were displayed in a barplot graph. (**e**) Gene ontology enrichment analysis for B cells performed and showed for both up and downregulated genes. (**f**) For GSEA analysis, targeted GOs from B cell subsets were targeted and showed. (**g**) naïve and memory B cells and their subsets are depicted, along with the up and down regulated genes involved in the suggested functional pathway. For all DEG analyses, a significant change was defined as an adjusted p-value (Benjamini-Hochberg analysis) less than 0.05 and a fold change over two times. For all GO and GESA analyses, an adjusted p-value (Benjamini-Hochberg analysis) of less than 0.05 was used to identify significant enrichment

B cell subsets showed two distinct heterogeneous clusters that were visualized using UMAP plot (Figure S3a). As shown in Fig. 1d, the total B cell population was not significantly different compared to untreated groups. However, the heterogeneity functional role of B cells and B cell subsets were identified by the significant changes in gene expression pattern (Figure S3b).Further, two heterogenous clusters such as naïve and memory B cells visualized using a UMAP plot were selected for in depth analysis (Fig. 3a). The population differences between the treated and untreated group exhibited minor differences in both naïve and memory B cells, together with an unidentified subset (Fig. 3b). Further, we used volcano plots to study DEGs associated B cell subsets that revealed significant differences via color differences. As a result, we identified more than 13,865 genes belonging to naive B cells compared to untreated groups. However, among them more than 96 genes were found to be significant, with 15 being upregulated and 81 being downregulated, as shown in Fig. 2c. Similarly, 11,930 DEGs were identified in memory B cells, with 46 being upregulated and 25 being downregulated (Fig. 3c). DEGs from B cell subsets are represented using a bar plot graph (Fig. 3d) that are distinguished by their up and downregulation expression patterns with an adjusted p-value. Both the B cells subset showed distinct expressions of DEGs, indicating heterogeneity in B cell function. In naïve B cells, *RPL36A, BASP1, LAMTOR1, OSBPL8, DNAJB14, RP11-138A9.1*, *CYF1P2, LINC0049, LPXN* and *CHST11* showed upregulation, while *SOD2, MSN, B4GALT1, C7ORF50, ELF2, EMC3, VAPA, LYST, GNG5* and *HIP1R* were downregulated respectively (Fig. 3d). In case of memory B cells, *SSR3, SNRPB2, NDUFA13, SPCS1, MAGED2, CRIP1, KDM2A, DNPH1, SPCS2* and *MDH2* were upregulated, while *IER2*, *MYC*, *TWISTNB*, *MAP11C3B*, *MIND*, *PASIP1*, *SIPA1L1*, *JMJD1C*, *SBDS*, and *BLOC1S4* were downregulated respectively (Fig. 3d).

A comparison of GO profiling of B cell subsets revealed biological, cellular, and molecular function of upregulated and downregulated genes, as shown in Fig. 3e and Figure S3d. Most of the upregulated genes are associated with memory B cells and most of them are involved in energy metabolism including precursor metabolites and energy, cellular aerobic respiration, protein localization, ATP metabolic process, oxidative phosphorylation, electron transport chain and protein targeting endoplasmic reticulum (Fig. 3e). Additionally, a few genes from memory B cells were involved in the downregulation of negative regulation of immune system process and monocyte differentiation (Fig. 3e). However, apart from cytoplasmic translation (upregulated), most genes from naive B cells were involved in downregulation of viral processing RNA splicing pathways such as mRNA processing, RNA splicing, regulation of mRNA metabolic process, and so on. GSEA was utilized to examine statistically significant and concordant changes between the treated and untreated groups. When exposed to AgNPs, B cells and their subset downregulated defense mechanisms such as innate immune response (Figure S3e, f). Among them, memory B cells showed significant GSEA score for various GO process including cell cycle (-0.539; p-value 0.019), immune response (-0.40; p-value < 0.001), defense response (-0.40; p-value < 0.001) and defense response to other organism (-0.40; p-value < 0.001) which demonstrated the downregulation of immunological response by the AgNPs treatment respectively (Figure S3e, f). Overall, as shown in Fig. 3g, the collective in-depth analysis from different approaches clearly distinguished functional heterogeneity of naïve and memory B cells with significant up and downregulation of DEGs with their proposed functional pathways.

### AgNPs treated T cells increased ribosomal RNA synthesis genes and inhibited T cell differentiation

**Fig. 4:**
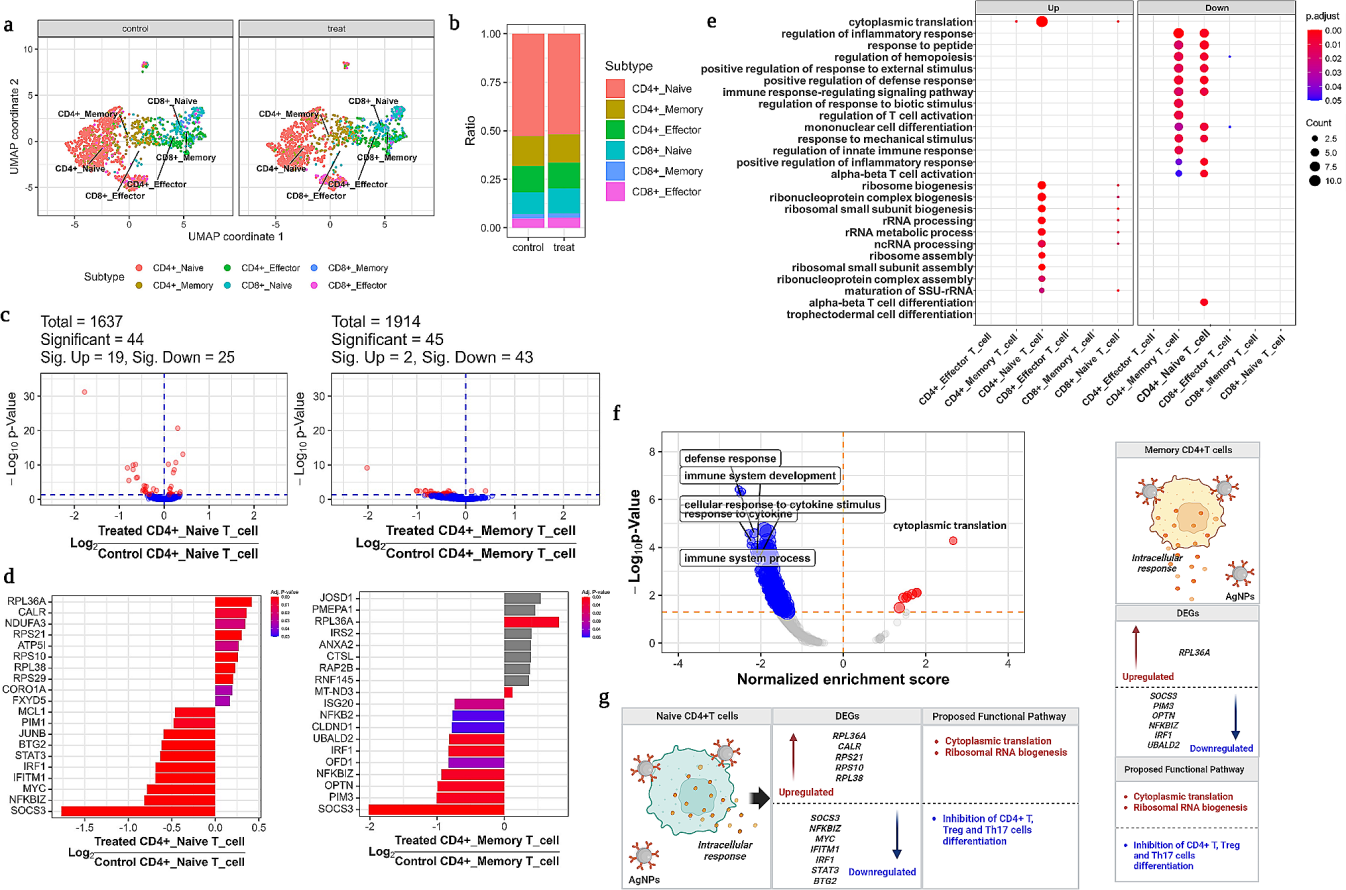
T cells subsets identification and functional annotation. (**a**) UMAP visualization of T cells subsets from CD4 + T and CD8 + T cells. (**b**) Comparison of cellular population from each susbset of T cells. (**c**) DEGs analysis of significant T cells subsets including naïve and memory CD4 + T cells were visualized in volcano plot. (**d**) Substantial genes expression from naïve and memory CD4 + T cells were represented in a bar graph based on log2 and an adjusted p-value. (**e**) Gene ontology functional annotation was performed for significantly expressed genes from each T cells subsets. (**f**) The enrichment analysis of significantly expressed genes from T cells is shown in a bubble plot based on gene ontology performance. (**g**) An illustration of the genes that are up- and down-regulated in the suggested functional pathway of naïve and memory CD4T cells. For all DEG analyses, a significant change was defined as an adjusted p-value (Benjamini-Hochberg analysis) less than 0.05 and a fold change over two times. For all GO and GESA analyses, an adjusted p-value (Benjamini-Hochberg analysis) of less than 0.05 was used to identify significant enrichment

T cells play a major role in adaptive immune response. They can act as “helpers” (CD4 + T cells) to support B cells to produce antibodies or “killers” (CD8 + T cells) to attack infected cells. Therefore, in this study, we compared the cellular and transcriptomic responses of CD4 + and CD8 + T cells, as well as their subsets, in the AgNP-exposed group to the untreated control group (Fig. 4; Figure S4). The cluster differentiation figure (Fig. 1b) depicted T cell heterogeneity and the functional role of T cells that might play an important role as a helper or cytotoxic effect in the AgNPs-treated group. Therefore, detailed profiling of total T cells is required to determine subsets and heterogeneity characteristics. Hence, we carried out further annotation to identify the subsets of total T cells from both groups, and the results revealed major subsets including naive, effector, memory CD4 + T cells and cytotoxic killer T cells, such as naive, effector, and memory CD8 + T cells, respectively (Fig. 4a). T cell subset population differences were observed and compared to the untreated group (Fig. 4b). Our results suggested that the effector and memory CD4 + T cells, as well as naive CD8 + T cells, showed a slight population increase but not significant, whereas other T cell subsets showed no differences.

Furthermore, DEGs analysis for total T cells were carried out to elucidate the genes that differed significantly. As a result, total 1637 genes were found to be expressed, of which 396 showed substantial expression, consisting of 28 upregulated and 368 downregulated genes (Figure S4a). Based on log2 and an adjusted p-value, the most significant genes were listed in a bar graph (Figure S4b). Likewise, we performed DEGs analysis for all T cell subsets to reveal unique gene expression from each T cell subset to scrutinize heterogeneity functional role (Figure S4c, d). Both naive and memory CD4 + T cells showed significant differences in gene expression (Fig. 4c). For naïve CD4 + T cells, 19 genes were upregulated, and 25 were downregulated among 44 genes, whereas in case of memory CD4 + T cells, 45 genes showed significant expression, among which 2 genes upregulated and 43 genes downregulated (Fig. 4c). There were no discernible differences among the other T cell subsets, such as effector CD4 + T cells, naive, effector, and memory CD8 + T cells (Figure S4c). Similarly, each T cells subset’s gene expression was represented in a bar graph based on log2 and an adjusted p-value (Figure S4d), and each one had a distinct list of genes that represented the heterogeneity among the T cells subsets. However, only naïve and memory CD4 + T cells showed substantial differences among the T cells, and the genes such as *SOCS3*, *NFKBIZ*, *IRF1*, *UBALD2,* and *RPL36A* showed similar expression in both the groups, while none of the other genes were expressed uniquely or revealed significant differences (Fig. 4d). For naïve CD4 + T cells, *SOCS3*, *NFKBIZ*, *MYC*, *IFITM1*, *IRF1*, *STAT3*, *BTG2*, *JUNB*, *PIM1*, and *MCL-1* genes expression was significantly downregulated, whereas *RPL36A*, *CALR*, *NDUFA3*, *RPS21*, *ATP51*, *RPS10*, *RPL38*, *RPS29*, *CORO1A,* and *FXYD5* genes were upregulated (Fig. 4d). In the case of memory CD4 + T cells, only two genes such as, *RPL36A* and *MT-ND3* were significantly upregulated while other genes including *SOCS3*, *PIM3*, *OPTN*, *NFKBIZ*, *IRF1* and *UBALD2* were substantially downregulated, respectively (Fig. 4d).

GO functional annotation such as biological process, cellular component, and molecular function were performed for significantly expressed genes from each T cell subsets. It has been shown that most of the downregulated genes from naive and memory CD4 + T cells were actively involved in the downregulation of immune defense response via inhibition of positive regulation of inflammatory response, that affect the regulation of hemopoiesis (Fig. 4e). Specifically, genes from memory CD4 + T cells downregulated T cell activation upon AgNPs exposure **(**Fig. 4e). Whereas upregulated genes from naïve CD4 + T cells participated in cytoplasmic translation and ribosomal RNA biogenesis including rRNA metabolic processing, ncRNA processing, ribosome assembly, and ribosome biogenesis (Fig. 4e). There is no significant gene participation from other T cell subsets that were observed.

The enrichment analysis of significantly expressed genes is shown in a bubble plot based on GO performance. Most of the genes involved in T cell activation were downregulated and actively involved in the inhibition of defense response upon AgNPs exposure including cytokine stimulating response, immune system development and process (Fig. 4f). Furthermore, some genes from both naïve and memory CD4 + T cells are involved in the downregulation of CD4 + T cells differentiation to avoid as a part of further adaptive mediates defense response. Whereas a few upregulated genes participated in cytoplasmic translation and others were involved in ribosomal RNA biogenesis that includes rRNA metabolic processing, ncRNA processing, ribosome assembly, and ribosome biogenesis (Fig. 4g).

## Discussion

### AgNPs interacted with metal ion to activate innate immune response

Monocytes are a type of circulatory immune cell that has a pattern recognition receptors (PRRs) that respond according to the external stimuli including the physicochemical characteristics or surface modification of nanoparticles [34, 35]. Previous study proved the activation of monocytes can lead to either subtype which can further influence innate and adaptive immune responses [36]. To reveal specific heterogeneity response of monocytes, we compared AgNPs treated and untreated groups that showed distinct role of monocyte subsets. Furthermore, monocyte subsets were identified by performing in-depth profiling, which identified CD16^+^ and CD16^−^ subsets **(**Fig. 2). The identified monocyte subsets CD16^+^CD16^−^ might induce high level of pro-inflammatory response by external stimuli [37], whereas some monocytes (CD16^−^CD16^+^) may facilitate phagocytosis, signal transduction, and degranulation by anti-inflammatory response respectively [38]. Our findings revealed a slight increase in the population of the CD16 + monocyte subset after AgNP exposure, though this increase may have inhibited inflammatory responses. Additionally, population reduction in monocyte subset CD16^−^ denoted the suppression of pro-inflammatory response upon AgNPs exposure **(**Fig. 2).

The DEGs from monocyte subsets also revealed the functional role of monocytes after AgNPs exposure. Notably, the upregulated MT genes *MT1G, MT1X, MT1E, MT2A*, and *MT1F* interacted with the metal ion in AgNPs to regulate ROS and suppresses oxidative stress. Several reports were published on MT genes, for instance, MT genes were actively interacted with the metal ion to regulate ROS and suppresses oxidative stress upon external stimuli such as chemical or heavy metals [39]. In another study, MT genes participated in metal ion detoxification, and free radical protection during oxidative stress [40]. Furthermore, leaching of Ag + from the surface of AgNPs via an oxidative process attributed to the upregulation of MT genes that trigger both extracellular and intracellular immunological responses [41]. In our study, though the cellular response to metal ion was activated by MT family genes, downregulation of *IL1β, TNFAIP8, CXCL10, CCL3,* and *CCL4* in both monocyte subsets denoted an inhibition of host-immune response towards AgNPs **(**Fig. 2). It has been proposed that MT genes participate in metal ion detoxification and suppress oxidative stress in response to AgNPs exposure. However, dose related toxicity of AgNPs needs to be addressed in future as MT may contribute to control oxidative stress probably depends on the ratio MT/Ag. As our study tested only one AgNPs dose, there is no evidence that a higher dose could not cause ROS due to MTs overload, and immune effects would exist must be further elucidated. Furthermore, macrophage inflammatory proteins such as MIP-1α (CCL3) and MIP-1β (CCL4) play a major role in immune responses against infection or inflammation in response to external stimuli [42]. Additionally, the interferon gamma-induced protein 10, also known as CXCL10, is involved in chemotaxis, apoptosis induction, and cell growth regulation in response to external stimuli [43, 44]. In our study, both monocyte subsets from the AgNPs-treated group downregulated CCL3, CCL4, and CXCL10 chemokines in response to AgNPs exposure, implying that AgNPs may avoid further immunomodulatory response even though cellular-metal ion association was highly modulated.

### B cell subsets interacted with metal ion but didn’t exhibit cytoxicity

Besides monocytes, B cells could also take part in innate immune response by modulating immunomodulatory functions. It also acts as a bridge between the innate and the adaptive immune responses by presenting the antigens to T cells [45]. However, the current analytical approaches have limitations to study the phenotype identification and functional role of B cells and therefore the functional role of B cells are still incomplete [46]. Therefore, our scRNA-seq analysis was used to identify distinct B cell populations that precisely categorize functional heterogeneity based on DEGs expression. Our study discovered two functional distinct populations of B cell subsets, naïve and memory B cells react with metal ions. In specific, in response to cellular association of AgNPs, naïve B cells showed regulation of colony formation, and cell proliferation. However, there is no further evidence to support the activation of B and T cell-mediated adaptive immune responses **(**Fig. 3). To support this finding, our study is the first to report the downregulation of SOD_2_ response in naïve B cells after AgNPs exposure demonstrating the inhibition of oxidative stress and avoidance of ROS generation **(**Fig. 3). Additionally, our findings were consistent with previous studies in which downregulation of *MSN* and *ELF2* (E74-like factor 2) helps to impair T and B lymphocyte proliferation and chemotaxis [46, 47], indicating that AgNPs do not influence toxicity in B cells. Further, Guan et al. [47]. and Wang et al. [48] reported, similar to our findings that the downregulation of *LYST*, *ELF2*, and *HIP1R* genes **(**Fig. 3) inhibited lysosomal trafficking which suppresses B and T cell mediated cytotoxicity [47, 49].

Our results were concordant with the previous report where activation of B cells were triggered by the binding of ligand which initiates an intracellular signaling leading to the internalization of antigen for processing and presentation to T cells [10]. The cellular association of AgNPs in memory B cells significantly inhibited further inflammatory response such as growth factors, and cytokines. The immediate early response gene 2 protein (*IER2*) and Myc proteins (*MYC*) genes play an important role in cell motility, cell matrix adhesion and in vitro capillary like structures formation by extracellular stimuli [50–52] were downregulated significantly to arrest further inflammatory response.

### AgNPs failed to activate adaptive immune response

The influence of AgNPs on adaptive immune responses is crucial but poorly investigated mainly due to the heterogeneous nature of the immune system as well as the limited capability of instruments [53]. Here, we investigated the AgNPs-related adaptive immune responses by analyzing the transcriptomic changes in T cell subsets, CD4 + and CD8 + T cells. Upon AgNPs exposure, both the naïve and memory CD4 + T cells, the genes *SOCS3* and *IRF1* were downregulated, which are important for CD4 + T cell differentiation [54, 55]. Furthermore, NF-B is a well-known inflammatory pathway mediator, and genes like *NFKBIZ* and *NFKB2* are primarily involved in the activation of both IL-17 A and TNF as chemo-attractants for neutrophil and monocyte recruitment [56]. In contrast to previous findings, our findings revealed that downregulation of both *NFKBIZ* and *NFKB2* contributed to the inhibition of inflammatory response. *MYC* and *IFITM1* genes were significantly downregulated suggesting the suppression of T cell-mediated responses.

## Conclusion

**Fig. 5:**
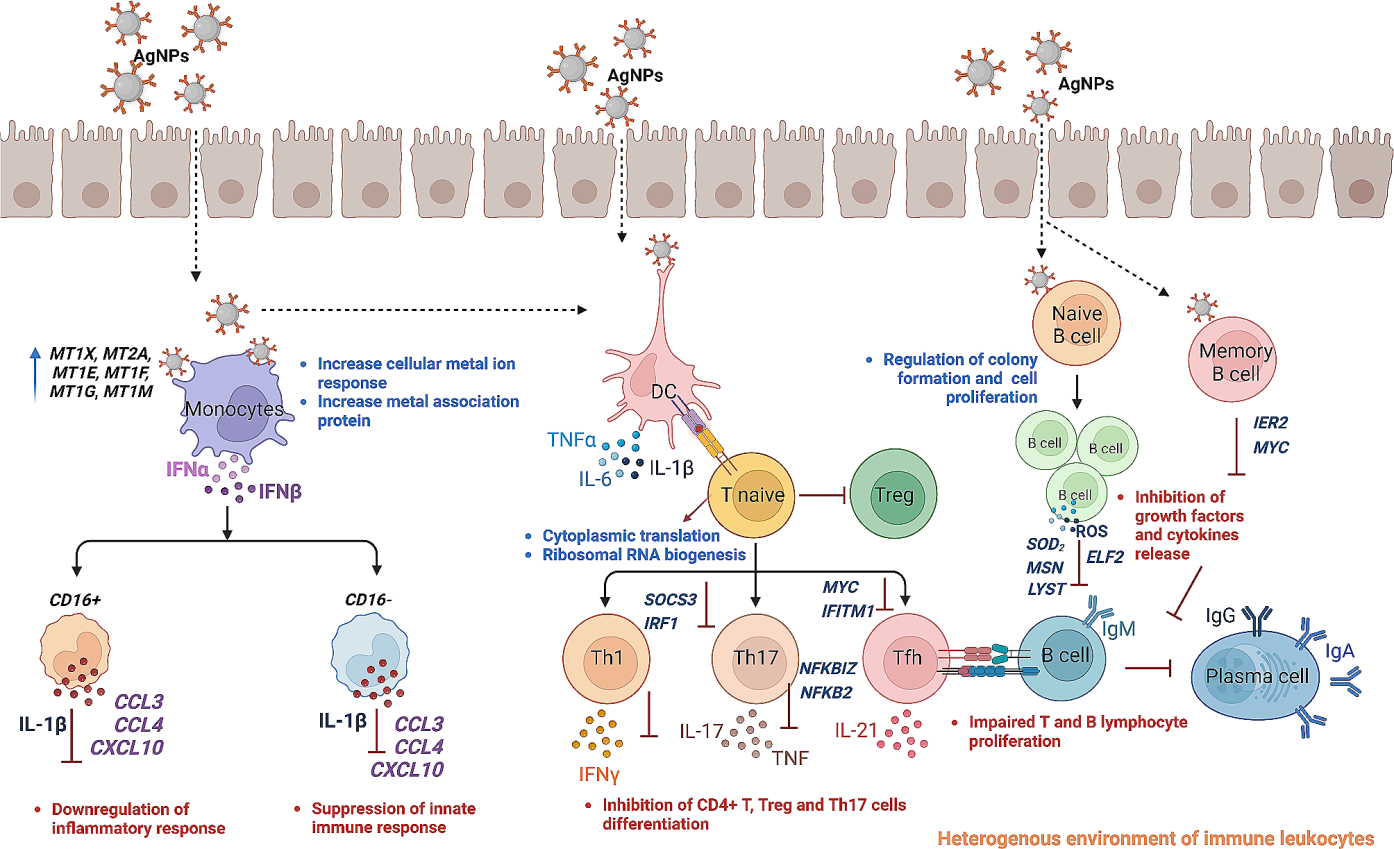
Proposed molecular mechanism of significant immune cells and their subsets upon AgNPs exposure

Our comprehensive scRNAseq analysis demonstrated the unique role of immune cell subsets such as monocytes, B cell, and T cell in suppressing immune mediated inflammatory response upon AgNPs exposure (Fig. 5). When AgNPs interact with monocytes there is an initiation of cellular-metal ion association which was confirmed by the upregulation of MT genes. Even though there is cellular-metal ion association, no significant activation of inflammatory response was observed. The association of AgNPs indirectly or directly with B cells increases cell proliferation by the regulation of colony formation. However, both B cells subsets have no cytotoxicity effect was confirmed by inhibition of oxidative stress and ROS generation. Furthermore, monocyte differentiation might downregulate DEGs from both naive and memory CD4 + T cells to prevent further initiation T cell mediated adaptive mediate immune responses as well as impaired B and T cell proliferation after AgNPs exposure. While scRNAseq allows us to investigate the immune response to AgNPs in a heterogeneous environment, this work alone is descriptive in nature, only partially conclusive, and requires further research to support our current conclusions. Additionally, our study tested only one AgNPs dose and there is no evidence that a higher dose could not cause ROS due to MTs overload, and immune effects would exist must be further elucidated. Overall, our results suggest that AgNPs did not show immune-related toxicity response in a diverse population, suggesting that more clinical research can be done to determine whether AgNPs are suitable for use in a safe manner.

### Electronic supplementary material

Below is the link to the electronic supplementary material.

Supplementary Material 1

Supplementary Material 2

Supplementary Material 3

Supplementary Material 4

Supplementary Material 5

Supplementary Material 6

Supplementary Material 7

Supplementary Material 8

## Data Availability

All the data of the current study are available from the corresponding authors upon reasonable request.
