# Supplementary figures and images for: scRNA-seq analysis discovered suppression of immunomodulatory dependent inflammatory response in PMBCs exposed to silver nanoparticles
Source: J Nanobiotechnology. 2024 Mar 17;22:118. doi: 10.1186/s12951-024-02364-0 (PMC10946150; doi:10.1186/s12951-024-02364-0)

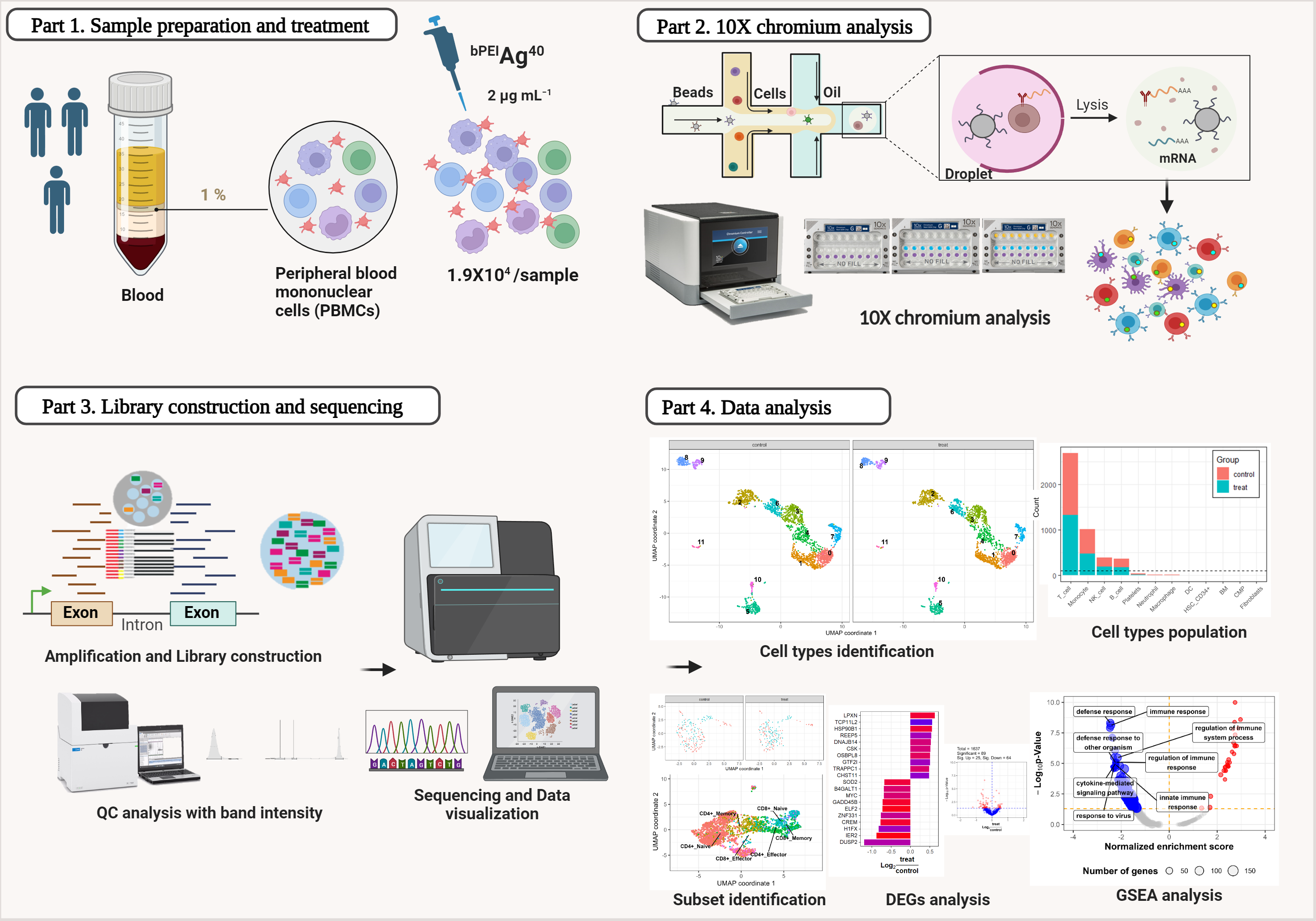

Supplement: Supplementary file 1 — Supplementary Material 1 [file 12951_2024_2364_MOESM1_ESM.png]
