# Supplementary material for: scRNA-seq analysis discovered suppression of immunomodulatory dependent inflammatory response in PMBCs exposed to silver nanoparticles
Source: J Nanobiotechnology. 2024 Mar 17;22:118. doi: 10.1186/s12951-024-02364-0 (PMC10946150; doi:10.1186/s12951-024-02364-0)
Supplement: Supplementary file 2 — Supplementary Material 2 [file 12951_2024_2364_MOESM2_ESM.docx]

**scRNA-seq analysis discovered suppression of immunomodulatory dependent inflammatory response in human leukocytes exposed to silver nanoparticles**

Haribalan Perumalsamy^1,2^, Xiao Xiao^2^, Hyun-Yi Kim^4^, Tae-Hyun Yoon^1, 2,3*^

*^1^Institute of Next Generation Material Design, Hanyang University, Seoul 04763, Republic of Korea*

*^2^Department of Chemistry, College of Natural Sciences, Hanyang University, Seoul 04763, Republic of Korea*

*^3^Department of Medical and Digital Engineering, Hanyang University, Seoul 04763, Republic of Korea*

*^4^NGeneS Inc.362, Gwangdeok 1-ro, Sangnok-gu, Ansan-si, Gyeonggi-do, Republic of Korea. 15495*

**Corresponding author**

Prof. Tae Hyun Yoon

Professor

Department of Chemistry

Hanyang University

Korea

E-mail: [taeyoon@hanyang.ac.kr](mailto:taeyoon@hanyang.ac.kr)


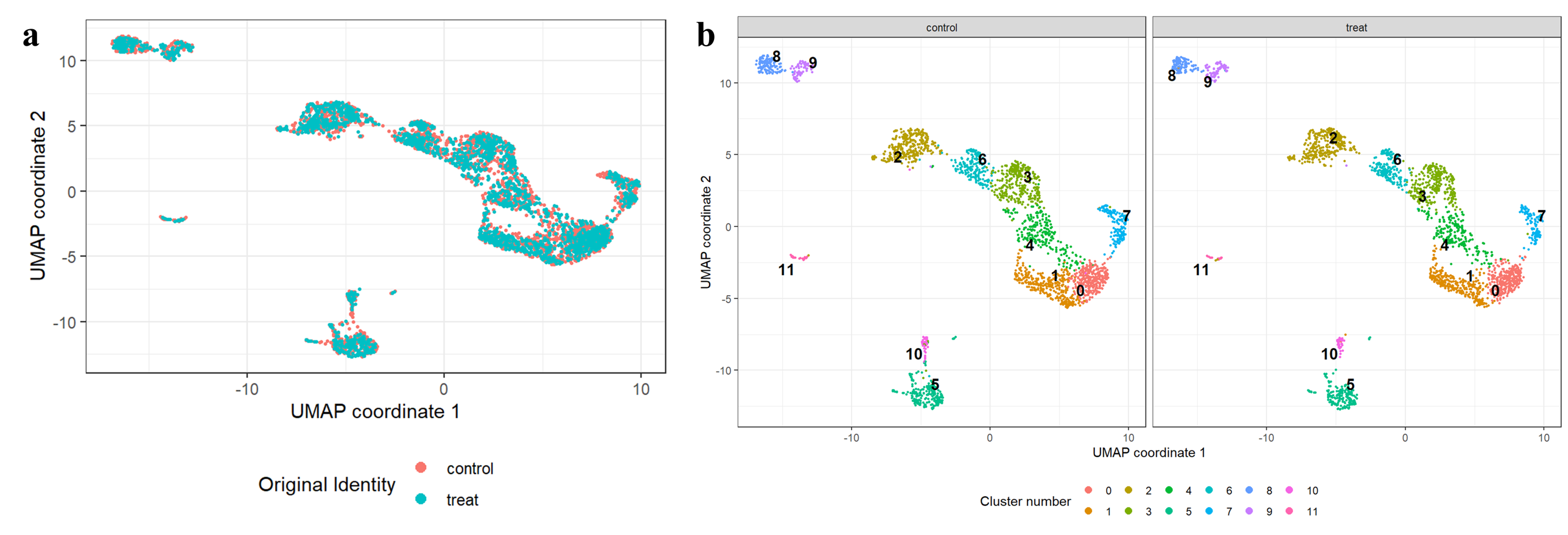


**Figure S1**. Comparison of population differences (a) and distinguished immune cell types clusters (b) between AgNPs hPBMCs treated and untreated control

**
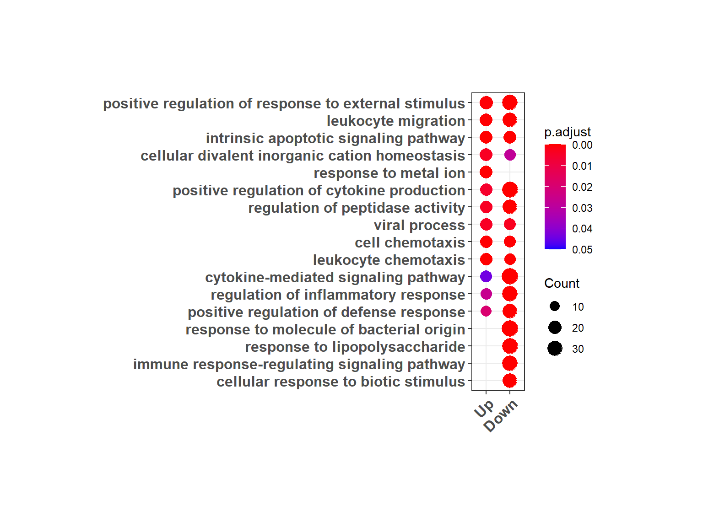
**


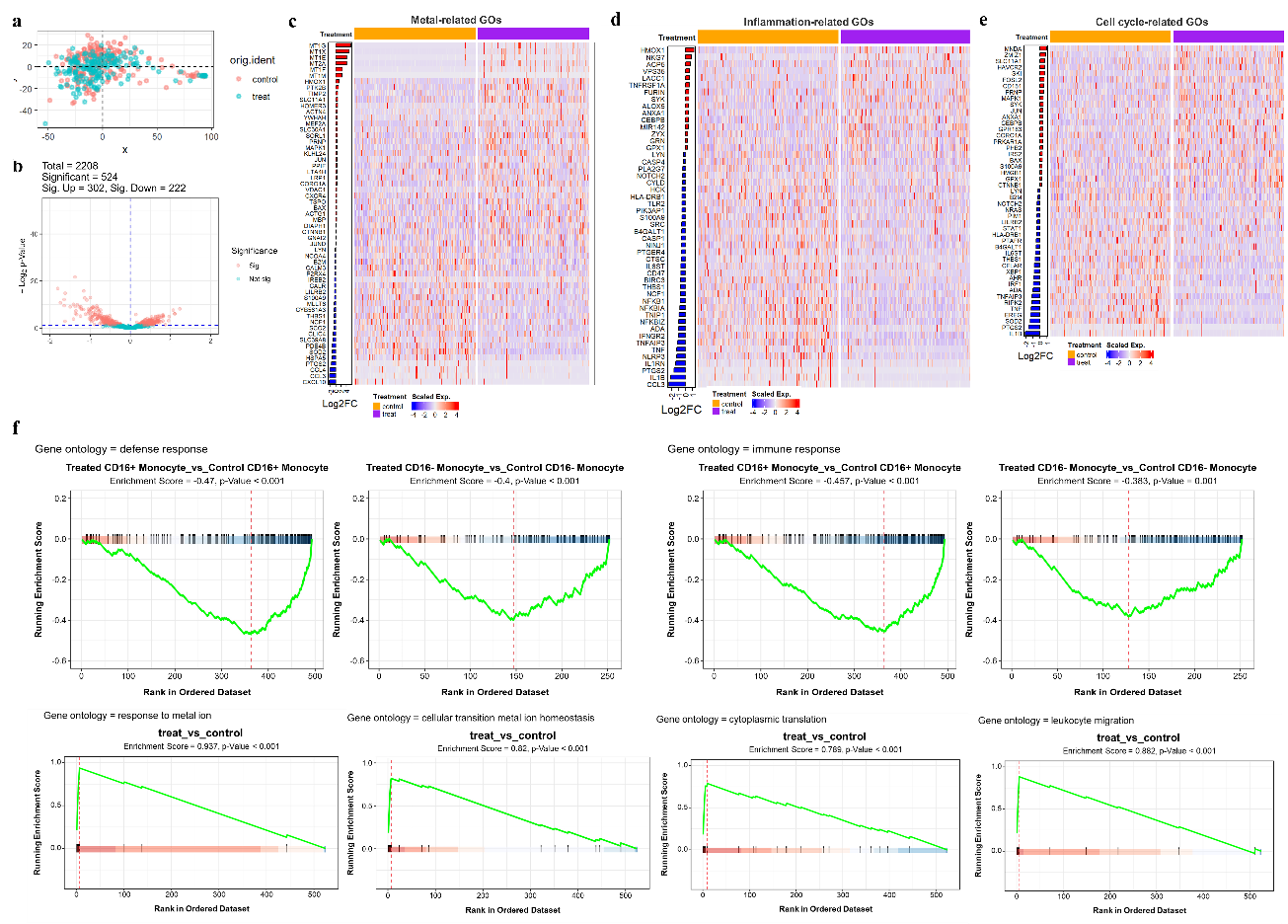
 **g**

**Figure S2**. **Comparison of total monocytes between AgNPs treated and untreated control and their functional enrichment analysis**. **a**. Expression of monocytes population between the groups; **b**. Volcano plots of DEGs from total monocytes; **c-e**. **f**. GSEA analysis of DEGs from total monocytes inflammatory response; **g**. Gene ontology term of total monocytes.


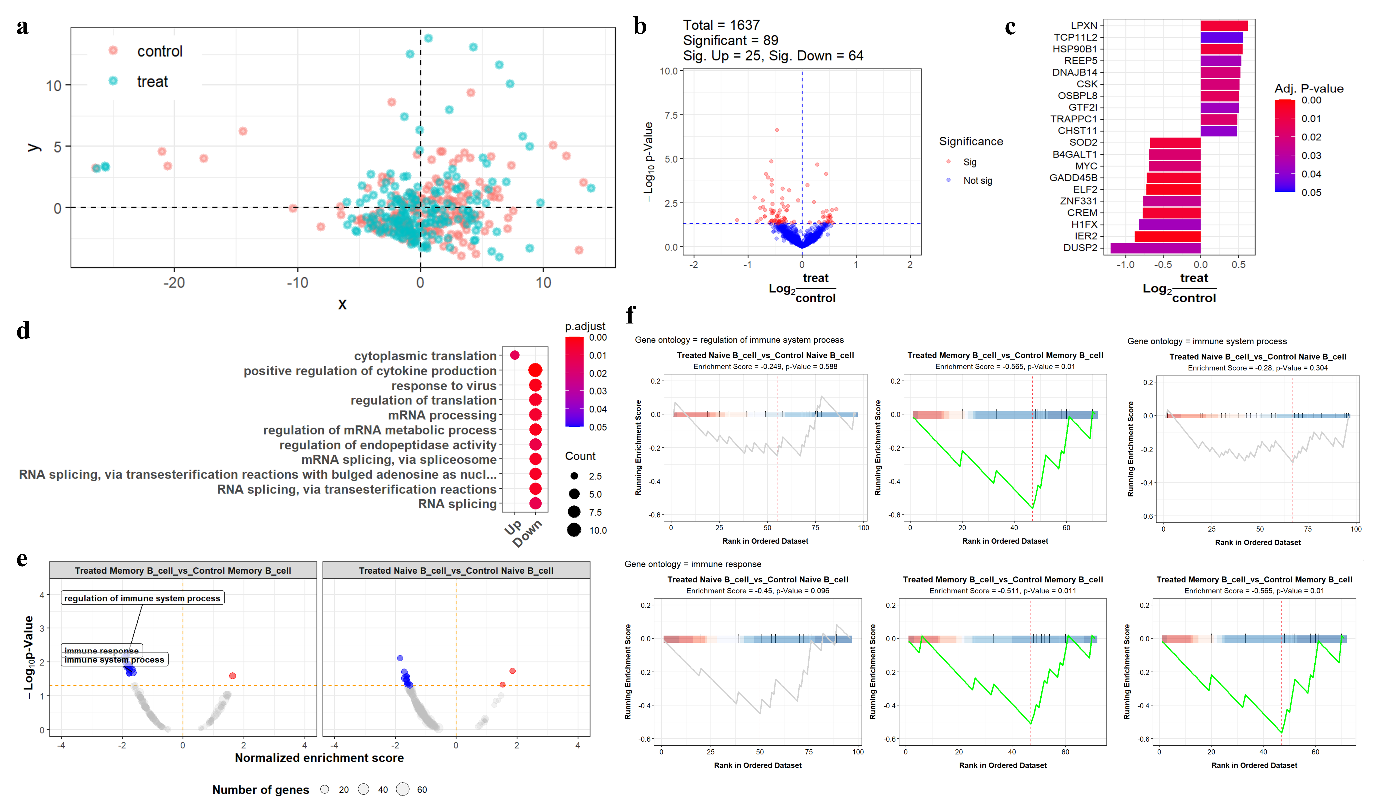


**Figure S3**. **Comparison of total B cells and their functional enrichment analysis.** **a**. Expression of B cells population between AgNPs treated and untreated control; b. Volcano plots of DEGs from total B cells; **c**. Bar blot diagram of DEGs from B cells; d. Gene ontology analysis of DEGs from total B cells; e. GSEA analysis of DEGs from total B cells **f**. GSEA analysis memory B cells with different parameter


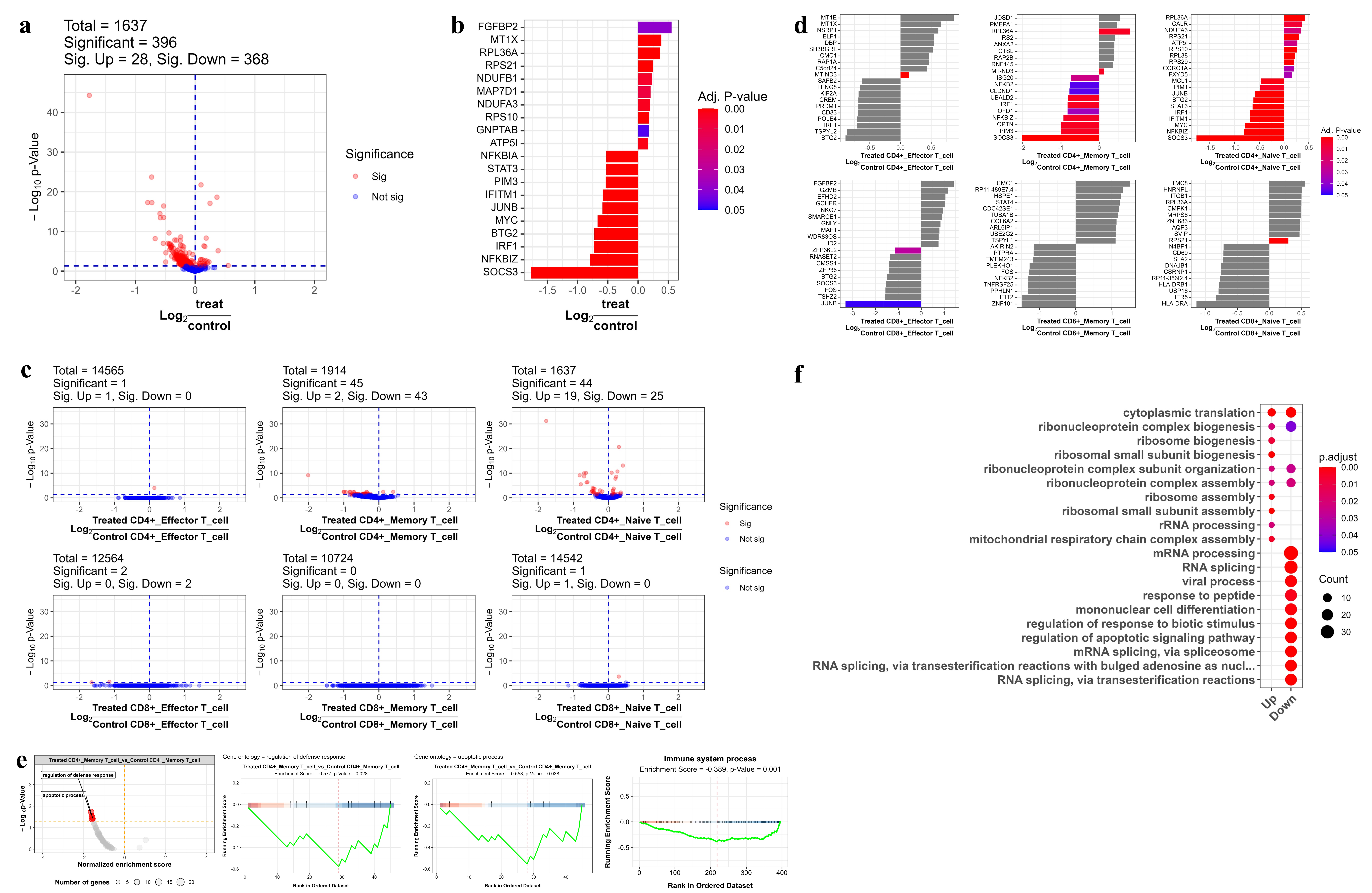


**Figure S4**. **Comparison of total T cells and their functional enrichment analysis.** **a**. Volcano plots of DEGs from total unbiased T cells; **b**. Bar blot diagram of DEGs from T cells; **c**. Volcano plots of DEGs from CD4+T and CD8+T cells. **d**. Bar blot diagram of T cells subsets including CD4+T and CD8+T cells. **e**. Gene ontology analysis of DEGs from total T cells. **f**. Gene ontology profile of total T cell
